# Do Consecutive Phacoemulsification Surgeries Under Topical Anesthesia Differ in Terms of Pain Perception and Cooperation?

**DOI:** 10.7759/cureus.19915

**Published:** 2021-11-26

**Authors:** Refika Hande Karakahya

**Affiliations:** 1 Ophthalmology, Lösante Hospital, Ankara, TUR

**Keywords:** visual analogue scale, phacoemulsification, pain perception, consecutive surgery, cataract

## Abstract

Background

Although intraoperative ocular pain has been investigated extensively in the literature, few studies have evaluated the pain perception between consecutive surgeries. Determining the facts about pain perception during phacoemulsification will allow surgeons to decide the type of anesthesia that best fits the patient when planning the contralateral cataract surgery. The aim of this study was to determine the level of pain perception, factors affecting pain perception, level of patient cooperation, and perception of operation time during consecutive phacoemulsification surgeries.

Methodology

This study included 314 eyes of 157 patients with bilateral senile cataracts who underwent phacoemulsification surgery under topical anesthesia with an interval of no more than six months. All patients underwent complete ophthalmic examination. Operation time, phaco time, surgeon’s comfort, and patient’s cooperation were recorded. Immediately after the operation, the patients graded the pain they experienced via the Visual Analogue Scale (VAS) from 0 to 10 and estimated the operation time.

Results

The mean VAS score was 0.88 ± 0.97 for the first eye and 1.50 ± 1.27 for the second eye (p < 0.011) surgery. The perception of the mean operation time was significantly lower in the first eye surgery (p < 0.001), even though the real objective operation time and phaco time were lower in the second eye surgery. The surgeon reported significantly more comfort during the first eye surgery. VAS was found to be positively correlated with nonsteroidal anti-inflammatory drug use, intraocular pressure, axial length, anterior chamber depth, central corneal thickness, phaco time, and operation time perception, and inversely correlated with best-corrected visual acuity and mature cataract morphology.

Conclusions

Consecutive phacoemulsification surgeries appear to differ not only in terms of pain perception but also operation time perception, patient cooperation, and surgeons’ comfort. Determining and controlling the factors that can influence patients’ pain perception and comfort will increase the safety of the contralateral surgery.

## Introduction

Phacoemulsification under topical anesthesia offers a safer alternative with a faster visual recovery time and higher patient satisfaction avoiding the potential risks of injection. However, the cooperation of the patient, which is closely related to the perception of pain, becomes essential for the surgeon’s comfort and better results of cataract surgery under topical anesthesia.

The clinical and experimental data highlight that pain perception differs among individuals according to their sex, race/ethnicity, and age. Women complain of more pain and have a lower pain threshold compared to men [1,2]. The role of age in pain perception is controversial. Some studies have reported that old age is a factor more sensitive to experimental pain, whereas others have advocated a lower sensitivity to pain with age [3,4]. In addition, pain perception may be influenced by the mood and psychological state of the patient. Severe anxiety, depression, and Type D personality are risk factors of severe postoperative pain [5]. In the case of phacoemulsification surgeries, studies have indicated that the perception of pain is influenced by gender, age, preoperative visual acuity [6], cataract type and stage of surgery [7], presence of diabetes mellitus, and longer duration of operation [8]. Considering consecutive cataract surgeries, there is an unproven but common belief among patients that the second cataract surgery is more painful. This is interesting because nearly similar procedures, similar steps in the same order, and similar manipulations are performed during the second surgery, with a similar operation time as long as no complications arise. There is no consensus regarding this information among the few reported studies with different designs [9-11]. However, determining the facts about pain perception during phacoemulsification will allow surgeons in deciding the type of anesthesia that best fits the patient when planning the contralateral cataract surgery. In this study, the level of pain perception, factors affecting pain perception, level of patient cooperation, and perception of operation time during consecutive phacoemulsification surgeries were investigated and compared.

## Materials and methods

Patient selection

This study conducted in the ophthalmology department of a tertiary university hospital included 157 patients with bilateral senile cataracts who were scheduled for phacoemulsification surgery under topical anesthesia. The study was conducted in accordance with the Declaration of Helsinki and was approved by the local ethics committee (Approval number: 2018-118). Written informed consents were obtained from all participants. Exclusion criteria included an allergic response to the anesthetic agent, hearing impairment, dementia or any other cognitive disease, involuntary movement disorders, anxiety disorders, eye movement disorders, keratoconus and anterior segment pathologies precluding vision peroperatively, posterior synechiae or other conditions requiring iris manipulations, iridophacodonesis, traumatic cataract, any disease likely to require posterior vitrectomy, any previous intraocular surgery, and an interval of more than six months between consecutive surgeries.

The eye with the lower best-corrected visual acuity (BCVA) was operated first unless the visual acuity was equal, in which case the patient made the decision. The timing of the second surgery was planned according to the patient’s complaints, requirements, and visual acuity level.

All patients provided their history of chronic diseases, smoking habits, alcohol consumption, and nonsteroidal anti-inflammatory drug (NSAID) overuse. Patients underwent a complete ophthalmologic examination. Intraocular lens, axial length (AL), central corneal thickness (CCT), and anterior chamber depth (ACD) were measured using a combined biometric pachymeter (PacScan 300AP Digital Biometric Ruler; SonoMed, Lake Success, NY, USA).

Anesthesia administration

All patients received topical anesthesia with 0.5% proparacaine hydrochloride (Alcaine 0.5%, Alcon Pharmaceuticals, Puurs, Belgium) drops applied to the ocular surface up to five times during a 10-minute interval before the operation. No sedation was used. Four patients who required additional intracameral anesthesia or subtenon injection were excluded.

Surgical technique

All procedures were performed by a single surgeon with experience of 15 years. All procedures were performed through 2.75 mm clear corneal incisions using Alcon Constellation (Alcon, Fort Worth, TX, USA) combined phacoemulsification-vitrectomy system by the same surgeon. Cataract extraction comprised continuous curvilinear capsulorhexis, hydrodissection and hydrodelineation, and phacoemulsification using the “stop and chop” or phacochop techniques, as well as aspiration of the cortical lens material followed by the implantation of a hydrophobic acrylic intraocular lens by the injector. After the ophthalmic viscoelastic substance was removed, the wound was sealed by corneal stromal hydration. Two patients whose wounds were sutured, three patients who required application of capsular tension ring due to zonular dehiscence, and one patient who underwent anterior vitrectomy for vitreous loss were excluded.

Assessment of pain perception and operation time perception

Immediately after the operation, the patients were asked to grade the pain they experienced on a Visual Analogue Scale from 0 (no pain) to 10 (unbearable pain) and to estimate the operation time following both operations. Following the second eye surgery, patients were also verbally asked which operation was more painful and which one took longer.

Assessment of perioperative patient cooperation and surgeon’s comfort

On completion of the surgery, the surgeon graded patients’ cooperation using a grading system (3: excellent; 2: good; 1: sufficient; 0: poor) and classified the operation as comfortable or uncomfortable.

Statistical analysis

Data analyses were performed using SPSS for Windows, version 22.0 (IBM Corp., Armonk, NY, USA). Whether the distributions of continuous variables were normal was determined by Kolmogorov-Smirnov test. Levene test was used to eveluate the homogeneity of variances. Data are expressed as mean ± standard deviation (SD) for continuous variables and number (percentage) for categorical variables. The differences in non-normally distributed variables among two dependent groups were analyzed by Wilcoxon test. Categorical dependent variables were evaluated with McNemar’s test. The degrees of relationship between variables was evaluated using Pearson or Spearman correlation analysis. P-values of <0.05 were considered statistically significant.

## Results

A total of 314 eyes of 157 patients who underwent phacoemulsification surgery under topical anesthesia were evaluated in this study. The male/female ratio was 89/68. The median age of the patients was 70.27 ± 10.34 years. The characteristics of the patients are shown in Tables 1, 2. The mean time elapsed between the first and second phacoemulsification surgery was 66.69 ± 42.88 days. A total of 26 (16.6%) patients were active smokers and seven (4.5%) patients were alcohol consumers. Overall, 43 (27.4%) patients had a history of NSAID use, and 27 (17.2%) patients used more than five tablets per month.

**Table 1:** Characteristics of patients undergoing phacoemulsification. Data are expressed as mean ± SD^†^  for continuous variables and number (percentage)* for categorical variables. NSAID: nonsteroidal anti-inflammatory drug; SD: standard deviation

		All cases (n = 157)
Age (year)^†^		70.27 ± 10.34
Gender (female/male)		68/89
Interval (days)^ †^		66.69 ± 42.88
Smoking habit^*^		26 (16.6%)
Alcohol consumption ^*^		7 (4.5%)
NSAID use^*^	1–5/month	27 (17.2%)
	>5/month	16 (10.2%)
Cataract type (First operation)^*^	Mature	40 (25.5%)
	Nonmature	117 (74.5%)
Cataract type (Second operation)^*^	Mature	7 (4.5%)
	Nonmature	150 (95.5%)

**Table 2:** Baseline preoperative characteristics. Data are expressed as mean ± SD^†^ for continuous variables and number (percentage)* for categorical variables. ^†^: Wilcoxon test; *: McNemar’s test; SD: standard deviation

		First operation	Second operation	P-value
Operation time^†^		10.52 ± 2.27	9.17 ± 1.20	<0.001
Phaco time^†^		0.44 ± 0.22	0.35 ± 0.13	<0.001
Visual Analogue Score^†^		0.88 ± 0.97	1.50 ± 1.27	<0.001
Operation time perception^†^		11.47 ± 4.65	12.78 ± 5.36	<0.001
Surgeon’s comfort^*^	Uncomfort	5 (3.2)	25 (15.9)	<0.001
	Comfort	152 (96.8)	132 (84.1)	
Patient cooperation^*^	Poor	2 (1.3)	7 (4.5)	<0.001
	Sufficient	5 (3.2)	13 (8.3)	
	Good	37 (23.6)	44 (28.0)	
	Excellent	113 (72.0)	93 (59.2)	
More painful^*^		22 (14)	87 (55.4)	<0.001
Longer^*^		26 (16.6)	86 (54.8)	<0.001

Ophthalmologic examination revealed mature cataract morphology in 40 (25.5%) eyes before the first eye surgery. Before the second eye surgery, seven (4.5%) eyes had mature cataracts (p < 0.001). The mean preoperative Snellen BCVA was 0.07 ± 0.07 and 0.13 ± 0.10 before the first and second eye surgery, respectively (p < 0.001). The mean intraocular pressure (IOP) values were 14.4 ± 4.08 mmHg and 14.63 ± 3.67 mmHg before the first and second eye surgery, respectively (p = 0.257). The mean AL was 23.71 ± 1.90 mm and 23.80 ± 1.89 mm before the first and second eye surgery, respectively (p < 0.001), whereas the mean preoperative ACD was 3.46 ± 0.53 mm and 3.48 ± 0.50 mm before the first and second eye surgery, respectively (p = 0.001). The preoperative CCT for both eyes was not significantly different (525.52 ± 29.41 vs. 525.95 ± 29.05 µm; p = 0.256).

The mean duration of operation was 10.52 ± 2.27 minutes for the first eye and 9.17 ± 1.20 minutes for the second eye surgery (p < 0.001). The mean phaco time was 0.44 ± 0.22 minutes for the first eye and 0.35 ± 0.13 minutes for the second eye surgery (p < 0.011). The mean VAS score was 0.88 ± 0.97 for the first eye and 1.50 ± 1.27 for the second eye surgery (p < 0.001). The mean operation time perception was 11.47 ± 4.65 for the first eye and 12.78 ± 5.36 for the second eye surgery (p < 0.001). The surgeon reported comfort in 152 (96.8%) operations in Group 1 whereas 132 (84.1%) operations in Group 2 (p < 0.001). In total, 22 (14%) patients stated more pain in the first operation, 87 (55.4%) patients reported the second operation as more painful, and the rest of the patients reported similar pain verbally (p < 0.001). In addition, 26 (16.6%) patients declared the first operation as longer, 86 (54.8%) patients reported the second operation as longer, and the rest of the patients reported a similar perception of duration verbally (p < 0.001). Patient cooperation was statistically significantly better during the first eye surgery (p < 0.001).

The VAS for the first eye surgery was positively correlated with NSAID use, IOP, AL, ACD, CCT, and phaco time, and inversely correlated with age, BCVA, and cataract morphology. The VAS for the second eye surgery was positively correlated with NSAID use, IOP, AL, ACD, and phaco time, and inversely correlated with BCVA (Table 3). The surgeon’s comfort for the first eye surgery was inversely correlated with alcohol consumption (p < 0.001). The surgeon’s comfort for the second eye surgery was positively correlated with BCVA (p < 0.001), CCT (p < 0.001), and immature cataract morphology (p = 0.047), and inversely correlated with NSAID use, IOP, and phaco time (p > 0.001). Patient cooperation for the first eye surgery was positively correlated with BCVA and inversely correlated with NSAID use, IOP, AL, ACD, and phaco time. Patient cooperation for the second eye surgery was positively correlated with CCT and inversely correlated with NSAID use, IOP, and phaco time (Table 4). Patient cooperation was statistically significantly better during the first eye surgery (p < 0.001), and was found to be correlated with the male sex in both surgeries (p = 0.003 for the first surgery and p = 0.001 for the second surgery).

**Table 3:** Correlation with Visual Analogue Score. NSAID: nonsteroidal anti-inflammatory drug; BCVA: best-corrected visual acuity; IOP: intraocular pressure; AL: axial length; ACD: anterior chamber depth; CCT: central corneal thickness

		Visual Analogue Score	
		First operation	Second operation
Smoking habit	r	-0.022	0.086
	p	0.788	0.282
Alcohol	r	0.078	0.133
	p	0.332	0.098
NSAID use	r	0.210	0.355
	p	0.008	<0.001
BCVA	r	-0.332	-0.290
	p	<0.001	<0.001
IOP	r	0.609	0.470
	p	<0.001	<0.001
AL	r	0.467	0.262
	p	<0.001	0.001
ACD	r	0.327	0.322
	p	<0.001	<0.001
CCT	r	0.176	0.027
	p	0.027	0.734
Cataract morphology	r	-0.202	-0.008
	p	0.011	0.917
Operation time	r	0.117	-0.116
	p	0.146	0.149
Phaco time	r	0.285	0.188
	p	0.000	0.018
Surgeon’s comfort	r	-0.112	-0.411
	p	0.161	<0.001
Patient cooperation	r	-0.447	-0.488
	p	<0.001	<0.001

**Table 4:** Correlation analysis for patient cooperation. NSAID: nonsteroidal anti-inflammatory drug; BCVA: best-corrected visual acuity; IOP: intraocular pressure; AL: axial length; ACD: anterior chamber depth; CCT: central corneal thickness

		Patient cooperation	
		First operation	Second operation
Smoking habit	r	-0.073	0.033
	p	0.361	0.678
Alcohol consumption	r	-0.062	-0.092
	p	0.443	0.252
NSAID use	r	-0.425	-0.458
	p	<0.001	<0.001
BCVA, Snellen	r	0.256	0.138
	p	0.001	0.084
IOP	r	-0.379	-0.290
	p	<0.001	<0.001
AL	r	-0.390	-0.128
	p	<0.001	0.111
ACD	r	-0.330	-0.074
	p	<0.001	0.356
CCT	r	-0.022	0.184
	p	0.781	0.021
Cataract morphology	r	0.166	-0.121
	p	0.037	0.132
Operation time	r	-0.089	-0.039
	p	0.266	0.630
Phaco time	r	-0.210	-0.275
	p	0.008	<0.001

Three patients in the first surgery group and one patient in the second surgery group with hard cataracts experienced corneal edema which resolved with medical treatment in a week. Four patients in the first surgery group and one patient in the second surgery group had raised IOP on the first operative day which responded to antiglaucomatous eye drops.

## Discussion

Cataract, the most common cause of preventable blindness, is commonly treated by phacoemulsification surgery globally [12]. Topical anesthesia has been accepted as a traditional method for phacoemulsification due to its rapid onset, ease of application, and high patient and surgeon satisfaction, along with avoidance of injection-related complications [13,14].

Intraoperative ocular pain during the surgery has been investigated extensively with variable results [6-11,15,16]. Sharma et al. [16] showed no statistically significant difference between consecutive topical surgeries under sedation. On the other hand, Bardocci et al. reported no difference for the consecutive cataract surgeries among the same population [9]. However, the small number of patients and female predominance who reported more inconsistent VAS scores between the operations may have affected their results. In this study, the VAS score and operation time perception were significantly higher for the second eye surgery than the first eye surgery. Despite shorter operation time, shorter phaco time, and shorter AL, patients graded the second operation with higher VAS scores. More than half of the patients verbally reported the second operation to be more painful (55.4%) and longer (54.8%) in duration, whereas 14% of patients reported more pain and 16.6% of patients reported a longer duration of operation for the first surgery. Interestingly, these were also reflected in the surgeon’s comfort and patient cooperation which were better in the first eye surgery. What causes this difference in pain perception during consecutive cataract eye surgeries, and how can it be eliminated for a more comfortable and safer surgery for both the patient and the surgeon?

The highest pain scores were reported to be during phacoemulsification as a part of cataract surgery [17]. Mobilization of the iris-lens diaphragm is the main cause of discomfort during phacoemulsification. Hou et al. reported that 45.2% of patients experienced pain when the anterior chamber was stretched by irrigation, which was decreased by aspiration or lowering the height of the infusion bottle [18]. Kang et al. reported the ocular parameters related to pain during phacoemulsification under topical anesthesia. Patients with higher baseline IOP as well as greater ACD and AL and IOP fluctuations during phacoemulsification experienced more perioperative pain [19]. In this study, female sex, young age, NSAID use, higher IOP, greater AL, greater ACD, thicker CCT, longer phaco time, poorer visual acuity, and mature cataract morphology were associated with more painful surgical experience. Thus, it may be reasonable to adjust lower bottle height before entering the anterior chamber or making a gentle hydrodissection, especially among long-term NSAID users, patients with longer globe suffering from lower scleral resistance, patients with higher baseline IOP, and patients having harder cataracts with low visual acuity. Moreover, informing the patient of the steps that may be painful can improve patient cooperation.

The additional controllable perioperative factor for perioperative pain is the duration of the surgery. Longer surgery was reported to be associated with greater pain in studies where the mean surgery duration was longer than our study [8,20,21]. In this study, the mean duration of operation was 10.52 ± 2.27 minutes for the first eye and 9.17 ± 1.20 for the second eye surgery. The objective operation time and patient-assessed operation time (operation time perception) (11.47 ± 4.65 vs. 12.78 ± 5.36 minutes) were significantly correlated (r = 0.186, p = 0.02) for the first eye surgery, which was not the case for the second eye surgery. However, we did not observe a positive association between operation time and pain score (r = 0.117, p = 0.146). This may be explained by the shorter operation time in our patients (median = 10 vs. 9 minutes). We observed more pain perception associated with longer phaco time. Therefore, measures to shorten the phaco time may help control the pain perception during phacoemulsification.

Younger age and female sex was associated with more pain perception in our study consistent with other studies where young age [22] and female sex [23] were linked to more pain. In addition, the male gender was determined to be significantly more cooperative during surgery.

Reducing preoperative anxiety can provide better operating conditions; therefore, preoperative instructions and detailed information about the surgery can reduce anxiety levels [24]. Unsurprisingly, patients are generally more anxious before their first surgery. Because the eye with a harder cataract is normally operated on first, higher expectations of success might increase the pain threshold of patients. Patients are generally more relaxed before the second eye surgery, and approximately half of the patients experience more pain during the second procedure [25,26]. Additionally, decreased anxiety during the second eye surgery may lead to increased awareness during the procedure, hence, lowering the pain threshold.

Cataract surgery, despite its duration and outpatient nature, may be an emotional stress factor triggering stress hormones [27,28]. This may, in turn, influence the memory of the first operation due to noradrenergic-glucocorticoid activity.

Diminished response to anaesthetic agent during the second procedure due to drug tolerance [29] and increase in monocyte chemoattractant protein-1 expression in aqueous humor in the contralateral eye suggesting a sympathetic ophthalmic-type uveitis [30] have also been proposed to explain the difference in pain perception during consecutive surgeries.

NSAID use, high preoperative IOP, poor visual acuity, longer phaco time, younger age, and female sex were found to be common factors affecting pain perception, surgeon’s comfort, and patient cooperation in our study.

The main limitation of the current single-center study was the small sample size. Further large-scale studies are needed to understand the pain management and underlying factors of pain perception during consecutive surgeries.

## Conclusions

The second phacoemulsification cataract surgery results in a more painful experience compared with the first surgery. Therefore, determining the factors that can influence patient’s pain perception and comfort, including NSAID use, high preoperative IOP, poor visual acuity, younger age, and female sex, will not only increase the safety of the surgery but also guide the surgeon in deciding the type of anesthesia that best fits the patient when planning the contralateral cataract surgery.

## References

[1] Fillingim RB, King CD, Ribeiro-Dasilva MC, Rahim-Williams B, Riley JL 3rd (2009). Sex, gender, and pain: a review of recent clinical and experimental findings. J Pain.

[2] Green CR, Anderson KO, Baker TA (2003). The unequal burden of pain: confronting racial and ethnic disparities in pain. Pain Med.

[3] Rittger H, Rieber J, Breithardt OA (2011). Influence of age on pain perception in acute myocardial ischemia: a possible cause for delayed treatment in elderly patients. Int J Cardiol.

[4] Lautenbacher S, Kunz M, Strate P, Nielsen J, Arendt-Nielsen L (2005). Age effects on pain thresholds, temporal summation and spatial summation of heat and pressure pain. Pain.

[5] Petrovic NM, Milovanovic DR, Ignjatovic Ristic D, Riznic N, Ristic B, Stepanovic Z (2014). Factors associated with severe postoperative pain in patients with total hip arthroplasty. Acta Orthop Traumatol Turc.

[6] Omulecki W, Laudanska-Olszewska I, Synder A (2009). Factors affecting patient cooperation and level of pain perception during phacoemulsification in topical and intracameral anesthesia. Eur J Ophthalmol.

[7] Apil A, Kartal B, Ekinci M, Cagatay HH, Keles S, Ceylan E, Cakici O (2014). Topical anesthesia for cataract surgery: the patients' perspective. Pain Res Treat.

[8] Dadacı Z, Borazan M, Öncel Acır N (2016). Pain perception in phacoemulsification with topical anesthesia and evaluation of factors related with pain. Turk J Ophthalmol.

[9] Bardocci A, Ciucci F, Lofoco G, Perdicaro S, Lischetti A (2011). Pain during second eye cataract surgery under topical anesthesia: an intraindividual study. Graefes Arch Clin Exp Ophthalmol.

[10] Yu JG, Ye T, Huang Q, Feng YF, Wang J, Fu XA, Xiang Y (2016). Comparison between subjective sensations during first and second phacoemulsification eye surgeries in patients with bilateral cataract. J Ophthalmol.

[11] Akkaya S, Özkurt YB, Aksoy S, Kökçen HK (2017). Differences in pain experience and cooperation between consecutive surgeries in patients undergoing phacoemulsification. Int Ophthalmol.

[12] Colin J, El Kebir S, Eydoux E, Hoang-Xuan T, Rozot P, Weiser M (2010). Assessment of patient satisfaction with outcomes of and ophthalmic care for cataract surgery. J Cataract Refract Surg.

[13] Morgan CM, Schatz H, Vine AK, Cantrill HL, Davidorf FH, Gitter KA, Rudich R (1988). Ocular complications associated with retrobulbar injections. Ophthalmology.

[14] Nicoll JM, Acharya PA, Ahlen K, Baguneid S, Edge KR (1987). Central nervous system complications after 6000 retrobulbar blocks. Anesth Analg.

[15] Kang YK, Kim MJ, Kim HK, Chun BY (2017). Clinical analysis of ocular parameters contributing to intraoperative pain during standard phacoemulsification. J Ophthalmol.

[16] Sharma NS, Ooi JL, Figueira EC (2008). Patient perceptions of second eye clear corneal cataract surgery using assisted topical anaesthesia. Eye (Lond).

[17] O'Brien PD, Fulcher T, Wallace D, Power W (2001). Patient pain during different stages of phacoemulsification using topical anesthesia. J Cataract Refract Surg.

[18] Hou CH, Lee JS, Chen KJ, Lin KK (2012). The sources of pain during phacoemulsification using topical anesthesia. Eye (Lond).

[19] Ulaş F, Balbaba M, Çelebi S (2013). Effect of prophylactic intraocular pressure-lowering medication on pain during cataract surgery. J Ocul Pharmacol Ther.

[20] Rothschild PR, Grabar S, Le Dû B, Temstet C, Rostaqui O, Brézin AP (2013). Patients' subjective assessment of the duration of cataract surgery: a case series. BMJ Open.

[21] Soliman MM, Macky TA, Samir MK (2004). Comparative clinical trial of topical anesthetic agents in cataract surgery: lidocaine 2% gel, bupivacaine 0.5% drops, and benoxinate 0.4% drops. J Cataract Refract Surg.

[22] Gombos K, Jakubovits E, Kolos A, Salacz G, Németh J (2007). Cataract surgery anaesthesia: is topical anaesthesia really better than retrobulbar?. Acta Ophthalmol Scand.

[23] Tan CS, Fam HB, Heng WJ, Lee HM, Saw SM, Au Eong KG (2011). Analgesic effect of supplemental intracameral lidocaine during phacoemulsification under topical anaesthesia: a randomised controlled trial. Br J Ophthalmol.

[24] Morrell G (2001). Effect of structured preoperative teaching on anxiety levels of patients scheduled for cataract surgery. Insight.

[25] Adatia FA, Munro M, Jivraj I, Ajani A, Braga-Mele R (2015). Documenting the subjective patient experience of first versus second cataract surgery. J Cataract Refract Surg.

[26] Nijkamp MD, Kenens CA, Dijker AJ, Ruiter RA, Hiddema F, Nuijts RM (2004). Determinants of surgery related anxiety in cataract patients. Br J Ophthalmol.

[27] Schelling G (2002). Effects of stress hormones on traumatic memory formation and the development of posttraumatic stress disorder in critically ill patients. Neurobiol Learn Mem.

[28] Hauer D, Kaufmann I, Strewe C, Briegel I, Campolongo P, Schelling G (2014). The role of glucocorticoids, catecholamines and endocannabinoids in the development of traumatic memories and posttraumatic stress symptoms in survivors of critical illness. Neurobiol Learn Mem.

[29] Ursea R, Feng MT, Zhou M, Lien V, Loeb R (2011). Pain perception in sequential cataract surgery: comparison of first and second procedures. J Cataract Refract Surg.

[30] Zhu XJ, Wolff D, Zhang KK, He WW, Sun XH, Lu Y, Zhou P (2015). Molecular inflammation in the contralateral eye after cataract surgery in the first eye. Invest Ophthalmol Vis Sci.

